# Data sharing: not as simple as it seems

**DOI:** 10.1186/1476-069X-10-107

**Published:** 2011-12-21

**Authors:** Neil Pearce, Allan H Smith

**Affiliations:** 1 Faculty of Epidemiology and Population Health, London School of Hygiene and Tropical Medicine, London, UK; 2 Centre for Public Health Research, Massey University Wellington Campus, PO Box 756, Wellington, 6140, New Zealand; 3 Division of Epidemiology, School of Public Health,, University of California, Berkeley, CA 94720, USA

**Keywords:** epidemiology, ethics, data sharing

## Abstract

In recent years there has been a major change on the part of funders, particularly in North America, so that data sharing is now considered to be the norm rather than the exception. We believe that data sharing is a good idea. However, we also believe that it is inappropriate to prescribe exactly when or how researchers should preserve and share data, since these issues are highly specific to each study, the nature of the data collected, who is requesting it, and what they intend to do with it. The level of ethical concern will vary according to the nature of the information, and the way in which it is collected - analyses of anonymised hospital admission records may carry a quite different ethical burden than analyses of potentially identifiable health information collected directly from the study participants. It is striking that most discussions about data sharing focus almost exclusively on issues of ownership (by the researchers or the funders) and efficiency (on the part of the funders). There is usually little discussion of the ethical issues involved in data sharing, and its implications for the study participants. Obtaining prior informed consent from the participants does not solve this problem, unless the informed consent process makes it completely clear what is being proposed, in which case most study participants would not agree. Thus, the undoubted benefits of data sharing does not remove the obligations and responsibilities that the original investigators hold for the people they invited to participate in the study.

## Background

In recent years there has been a major change on the part of funders, particularly in North America, so that data sharing is now considered to be the norm rather than the exception. Data sharing is clearly, in general, a good idea. However, it is not as simple as it seems. The devil is in the detail, and the detail is highly specific to each study, and each potential data recipient. In this paper, we will therefore discuss some of the concerns and caveats which should be taken into account in any data sharing policy, or any individual decision about data sharing.

More than twenty years ago, one of us (NP) was involved in a series of studies that identified a beta agonist asthma drug (fenoterol) as the cause of an epidemic of asthma deaths in New Zealand [1]. The accuracy of the data was disputed by other researchers, so prior to publication we organised several reviews, conducted blind, where they sat down with us and reviewed the general practitioner questionnaires and the hospital records (which were the basis for the drug prescribing data); this found that the classification of the data had been accurate [2]. However, following publication, the study findings were strongly disputed by the pharmaceutical company involved [2], and by an 'expert panel' which was assembled by the company [3]. The company requested the raw data for the studies, using the New Zealand Official Information Act, which applies to universities.

We checked with the regulations of the New Zealand Medical Research Council, our main funder, and these stated that data could be shared with other 'bona fide researchers', but the decision was to be left up to the researchers who had originally collected the data - it was they who should decide who were 'bona fide researchers' who the data could be shared with. Since we did not consider the company, or its hired consultants, to be 'bona fide researchers' - in fact, we felt they had a vested interest in the issue, and had seriously misrepresented the published findings of our studies [4-6] - we were not inclined to give them a copy of the data. Furthermore, we were concerned about confidentiality, since New Zealand is a small place, and study participants could potentially be identified on the basis of demographic data, even if the data set was 'anonymised'. Also, we considered that it was inappropriate for the company to be given the data without specifying in advance what analyses it intended to conduct.

We offered the company a solution: it could specify in advance what its planned analyses were, and someone nominated by the company could then visit our research group, and we would do the analyses with them, without handing over a copy of the data. The company was unwilling to accept this option, and persisted with its claim for the data under the Official Information Act. We therefore referred the matter to the Ethics Committees that had originally approved the study. All but one of them ruled that the company should not be given the data - the other Committee was unwilling to make a ruling, but recommended that the Company should apply directly to them, and they would consider the request. We also referred the issue to the New Zealand Ombudsman, who has jurisdiction over Official Information Act requests. After reviewing the issue in detail for several months, the Office of the Ombudsman ruled that the data should not be given to the company, on the grounds that it was impossible to protect confidentiality, even if personal identifiers such as name and date-of-birth were removed.

More recently, one of us (NP again) was involved in another dispute about data sharing, from the other side. He was involved in producing a report on the health status of the population of the Pacific Island country of Tonga, with funding from the World Health Organisation (WHO) [7]. Surveys had been conducted, by a group of Australian researchers, in 2004, as part of the WHO STEPwise approach to Surveillance (STEPS) project, but few of the findings had been published, and the researchers were apparently unwilling to make the data available to the Tonga Ministry of Health. Our report noted that:

"The Tonga STEPS study... has collected very useful data on [non-communicable diseases] NCDs and their risk factors. Participation in similar such surveys is important to ensure the continuation of accurate determination of disease prevalence in Tonga. However, it is a concern that little of the STEPS data has been published (four years after it was collected), and of the materials published little is available to the Ministry of Health. The raw data set has not been made available to the Tongan Ministry of Health, or to the authors of this report. It is essential that in future, a copy of such data is made available to the Tongan Ministry of Health. Otherwise, such surveys will be of little benefit to the health of Tongans."

In this instance, we were attempting, on behalf of the Tonga Ministry of Health, to obtain access to a data set collected (with cooperation and assistance from the Ministry of Health) by a group of university-based researchers. A couple of years ago, one of us (NP) was involved in such a dispute from the other side, where his university-based research group was denied access to a data set collected by the New Zealand Ministry of Health. Our group had produced a series of monitoring reports, funded by the Ministry of Health, on the New Zealand National Cervical Screening Programme (NCSP) [8-11]. The programme was established in 1991, and ever since had been dogged by adverse publicity [12]. Our own reports had highlighted ethnic differences in screening access and uptake; our contract was terminated after four years. Independently of that, we were conducting a programme of research into demographic differences in cervical cancer survival, with a particular focus on ethnic differences [13-15]. We managed to obtain access to a NCSP data set for a small number of cervical cancer cases for one particular set of analyses [14], but our request for further access to the Registry data, in order to investigate ethnic differences in screening, and how these had changed over time, was denied, even though the study was funded by the Health Research Council (HRC) of New Zealand, had received ethical approval, and had also received approval from the National Kaitiaki Group (a Maori Committee which exercises guardianship over the Maori data in the Registry), and the Pacific Women's Advisory Group. Confidentiality was one of the key reasons given for refusing access to the data, despite all of the necessary approvals having been obtained; one can only speculate whether the possibility of further adverse publicity about the shortcomings of the screening programme may have also played a role.

These three incidents, over two decades, illustrate the hazards and benefits of data-sharing. In the two decades since the fenoterol studies were published, there has been a major change on the part of funders, particularly in North America, so that data sharing is now considered to be the norm rather than the exception. In fact, it would be a brave researcher nowadays who declared themselves to be against data sharing - they would generally be regarded as having something to hide, and would be unlikely to obtain further funding (just in case any of our potential funders are reading this, let us assure them that we are not against data sharing under appropriate conditions!). On the other hand, there has been increased concern about confidentiality and access (by researchers) to patient records [16]. So access (by other researchers) to data collected by researchers has perhaps become more easy, whereas access (by researchers) to data collected by governments has in some cases become more difficult.

### Options for data sharing

The usual options for data sharing were outlined in a recent Request For Information from the National Institutes of Health (NIH) regarding strategies to encourage broad data sharing in environmental health sciences research [17]. The options include: (i) a study investigator responding ad hoc to individual requests for data; (ii) establishing informal collaborative networks among investigators with common interests to conduct focused analyses (e.g. data pooling); and (iii) regular deposits of study data in a data enclave or externally-managed public archive.

The options for data sharing were also recently reviewed by Reiter and Kinney [18^]^. They noted that simply stripping a data set of unique identifiers such as names, addresses, and identification numbers may not suffice. For example, Sweeney showed that 97% of records in voter registration lists for Cambridge, MA, could be uniquely identified using birth date and 9-digit zip code. In fact similar methods have been used on census data in New Zealand to create cohorts that can be followed over time, and linked with cancer and death registration data [19,20].

Reiter and Kinney classified data sharing into restricted-access strategies, and restricted data strategies.

Restricted-access involves sharing unaltered confidential data with external researchers while preserving confidentiality. This can be achieved by: (i) providing a copy of the data under a license agreement to researchers with legitimate research questions and (possibly) destroyed at project completion; or (ii) researchers may be required to carry out their analyses in a physically and electronically secure facility controlled by the data steward, and may be required to first submit a research proposal.

Restricted-data strategies involve unfettered access to data that have been modified before release. The key issue is how much information should be deleted or transformed to ensure data protection without destroying the usefulness of the data [18^]^. Risks can be reduced by altering data, e.g. by coarsening, data swapping, noise addition, or synthetic data [18].

It is noteworthy that none of these proposed data-sharing strategies involves simply making the data set available to anyone who requests it. Data-sharing is viewed as a matter of negotiation rather than compulsion. Furthermore, most approaches rely on a 'data steward' (usually the researchers who collected the data) who decides who should have access to the data (i.e. who is a 'legitimate researcher' or who has a 'legitimate research question') and in what form it should be made available.

### Funders

Funders naturally like data sharing because it appears to make better use of the resources that they have allocated for research. The NIH Statement on Sharing Research Data states that "NIH reaffirms its support for the concept of data sharing. We believe that data sharing is essential for expedited translation of research results into knowledge, products, and procedures to improve human health. The NIH endorses the sharing of final research data to serve these and other important scientific goals" [21]. More recently input has been sought by the National Institute for Environmental Health Sciences (NIEHS) on "strategies to encourage broader data sharing among researchers in the field of environmental health science who are conducting clinical or epidemiologic studies."[17] The United Kingdom-based Wellcome Trust website states that "we aim to ensure that the data generated by the research we support is managed and shared in a way that maximises the benefit to the public." Similarly the United Kingdom Medical Research Council (MRC) website states that:

Our policy builds on the central principles of the Organisation for Economic Co-operation and Development (OECD) in its report "Promoting Access to Public Research Data for Scientific, Economic and Social Development". These are that publicly-funded research data are a public good, produced in the public interest, and that they should be openly available to the maximum extent possible... Our data sharing and preservation policy applies to all MRC-funded research. It does not prescribe when or how researchers should preserve and share data, but requires them to make clear provision for doing so when planning and executing their research.

Most other similar funding bodies have now adopted similar policies, i.e. they do not prescribe how data should be shared, but they require that applicants for funding have a plan for doing so.

### Journals

Some journals (but few epidemiology journals to date), are now also requiring data-sharing. Journal policies were recently reviewed by Alsheikh-Ali et al [22]. They found that of the 50 original research journals with the highest impact factor, 44 (88%) had a statement about data sharing; there was a wide variety of journal requirements, ranging from requiring the sharing of all primary data to just including a statement in the published manuscript stating that data can be available on request. Of 500 assessed papers, 149 (30%) were not subject to any data availability policy; of the remaining 351 papers, 208 (59%) did not fully adhere to the data availability instructions, most commonly (73%) by not publicly depositing microarray data. It is notable, however, that the majority of papers considered were molecular research, which would carry much less (or often zero) issues of confidentiality compared with epidemiological/observational studies, which were underrepresented in the sample [22]. This is an important issue for epidemiologists, since journal policies may evolve with a focus on molecular research, where mandatory data sharing does not raise the same ethical issues.

Recently, the publishers of this journal (BMC) issued a draft position statement on open data (http://blogs.openaccesscentral.com/blogs/bmcblog/resource/opendatastatementdraft.pdf) which was strongly in favour of data sharing, but stated that:

The decision to mandate data deposition as a condition of publication is another decision best made by the scientific community concerned rather than a single journal or publisher... We will, therefore, support data publication when it is mandated, but will also enable, encourage and recognize data sharing and publication on a voluntary basis for scientists wishing to show leadership in their field.

A corollary of this approach is that it would be useful to have a statement in published manuscripts, similar to the Competing Interests Statement, that sets out the degree of availability or openness of the data.

### The investigators

Researchers, understandably, may have more mixed feelings about data sharing. This usually stems from two main concerns.

Firstly, there is the question of 'ownership'. It is understandable that researchers may feel unhappy if they work for several years to develop a research proposal, get the funding, get the ethical approval, hire staff, collect the data, clean the data, and produce the first publication, to then have the data quickly "shared" with investigators who have done none of this work. This is not a trivial issue, since a single study may produce a whole series of publications, but data may be required to be shared as soon as the first publications in the series have appeared. For example, the NIH statement of sharing research data states that "NIH recognizes that the investigators who collect the data have a legitimate interest in benefiting from their investment of time and effort. We have therefore revised our definition of the timely release and sharing to be no later than the acceptance for publication of the main findings from the final data set"[21]. The wording used does not define "main findings" and presumably allows for the fact that the main findings from a study may be published in several papers over some years.

These issues were at the core of a recent dispute in which data on sperm counts in Denmark were published without the permission of the researchers who had collected it [23]. The researchers in question were required to supply the data as part of internal reports to the Danish National Board of Health, who then posted a graph online, which was then incorporated into a paper published in Epidemiology [24]. An accompanying editorial [25] noted that "the presentation of a few raw data on a Web site - or in a commentary - is hardly the preferred way to advance science. But neither is it acceptable for valuable data to be held in storage. The publication of these data in *Epidemiology *does not foreclose the opportunity for researchers to prepare a full and careful analysis of their data."

It is clearly not in the public interest to have data collected for research projects funded by public money to be lying unpublished for many years. However, it is also clearly not in the public interest for researchers to feel that it is not worth collecting the data in the first place, and that an easier path to publication, and scientific glory, is simply to regularly request access to data that colleagues have collected. Both extremes are not in the interests of science, or public health. A balanced approach is required in which data is made available, to genuine researchers with a genuine research question, once the initial investigators have had an adequate time to publish their main findings.

The second issue that is usually of major concern to the researchers who collected the data is usually the possibility of the data being obtained by hostile agencies with vested interests in the outcome of the study. Such disputes have perhaps become more frequent in recent years [26]. The usual approach is for the company concerned to hire consultants to criticise the research publicly, either when it appears in print, or even prior to publication [27]. In recent years, these efforts have been further developed and refined with the use of websites and publicity that stigmatizes unwelcome research findings as "junk science" [28]. In some instances these activities have gone as far as efforts to block publication [2]. Recent examples include attempts to influence studies on the toxicity of benzene [29] and diesel particulate matter [30], the various industry efforts over many years to influence the conduct and interpretation of research into the health effects of dioxin [31], the industry campaign to undermine an Occupational Safety and Health Administration (OSHA) chromium (VI) standard [32] and corporate infiltration of a panel convened to set standards for chromium (VI) in California [33]. More recently, epidemiology in general, and occupational epidemiology in particular, has been criticised for a inherent tendency to produce false positive findings [34], a view which has been disputed by other epidemiologists including one of the authors (NP) [35]. It is in this context that, at least from some quarters, demands for data-sharing have arisen, e.g. with respect to industry attempts to dispute the findings of studies of health effects of air pollution in the United States [36].

As one of us (NP) commented recently, "for every independent epidemiologist studying the side effects of medicines and the hazardous effects of industrial chemicals, there are several other epidemiologists hired by industry to attack the research and to debunk it as 'junk science'."[37] Compulsory data-sharing with such 'hired guns', particularly when the primary researchers have not had the full chance to publish their findings, creates even more disincentives to collect primary data in the first place. This is bad for everyone, including the 'hired guns' who will have no contracts if independent researchers are no longer collecting primary data which they can subsequently critique.

### The study participants

So who is missing from this debate? The study participants! It is striking that most discussions about data sharing focus almost exclusively on issues of ownership (by the researchers or the funders) and efficiency (on the part of the funders). There is usually little discussion of the ethical issues involved in data sharing, and its implications for the study participants, apart from the requirement that (usually) participants are not able to be identified from the data set that is shared.

Obviously the issues involved differ according to the way that the data was collected, and the information that was collected. For example, sharing of routine death certificates, or even cancer registrations, carries quite different ethical considerations to that of sharing data that was collected by an individual researcher (or group) for a specific study, in which informed consent was obtained. The response to this dilemma from many funding agencies is simply that the informed consent process should include consent to the data being shared. However, our experience is that most study participants would not fully understand what was involved in such a request, and if they did they would probably say "no". We therefore consider that it is unethical for us to request study participants to sign an 'informed consent' that we would not sign ourselves.

It's one thing to consent that "the researchers may carry out further analyses of the data including analyses of additional health outcomes and/or analyses which involve colleagues from other researcher groups". It's completely another issue to agree that 'anyone, even companies with vested interests, or members of the general public, will be able to obtain a copy of the data from this study, with the names and dates of birth removed'. Requiring such 'genuinely informed consent' would see most epidemiological research grind to a halt, which is in the interests of neither researchers, funders, the general public, or science itself.

These issues are particularly acute in community-based studies, e.g. of occupational or environmental exposures and their health effects. Usually, the Principal Investigator and the Co-investigators will be named on the consent form, which may also explicitly list which other researchers will have access to the data. Prior to ethical approval, and funding, being obtained, the researchers may hold a series of meetings with community leaders and potential participants - this is particularly the case in countries like New Zealand where research may involve indigenous communities [38]. Usually, the communities concerned will only give consent for a study to proceed once they trust the researchers, and are confident that the data will be used carefully, and will not be shared with other researchers unless the main research group retains control over, and responsibility for, the way that the data is used and reported. Researchers may also be required to go through many other 'hoops' to gain ethical approval, including assurances about how the data will be stored, how confidentiality will be maintained, how the findings will be reported back to study participants, and who will be consulted before the findings are published.

The problem with compulsory data sharing is that it completely bypasses these necessary checks and balances. In the most extreme case, other researchers may simply be able to 'take' data that has been collected under very stringent conditions, and use it any way that they wish, without having to go through any form of ethical approval, and without specifying their research protocol in advance. In this extreme situation, data sharing raises very significant ethical concerns, which go way beyond the needs of funders, researchers, or the scientific community.

And how should informed consent be obtained for such data sharing? If the consent is "informed" it needs to be explained. Here is an example of what might be needed:

This study is being directed by Professor xxx of the University of yyy. The study is being conducted because preliminary findings suggest that arsenic concentrations in your drinking water may be harmful to your health. More information is needed to confirm this, and it is for this reason we are inviting you to participate in our study. If we do find scientifically valid evidence of increased health risks, it is possible that the arsenic drinking water standard may be reconsidered and lowered by the Environmental Protection Agency (EPA). However, the National Institutes of Health (NIH) which funds our work requires that we share the data with others, even if they refuse to collaborate with us. This includes mining companies, who believe the current drinking water standard is already too low. Runoff water from the mining properties has to meet drinking water standards, and the mining companies do not wish to pay for the costs of further cleanup. They have already hired consultants who have been opposed to our previous work. Your name and all personal identifiers will be removed from any data given to them, but you may see publications from this study by the mining companies or their consultants who already claim that there are no increased health risks from arsenic in your water. Sorry about that, but please sign this form anyway".

## Discussion

So what is the solution? We believe that data sharing is a good idea, and the onus should be on researchers to share their data with other researchers once the main findings have been published. However, we also believe that it is inappropriate to prescribe exactly when or how researchers should preserve and share data, since these issues are highly specific to each study, the nature of the data collected, who is requesting it, and what they intend to do with it. The level of ethical concern will vary according to the nature of the information, and the way in which it is collected - analyses of anonymised hospital admission records may carry a quite different ethical burden than analyses of potentially identifiable health information collected directly from the study participants.

Furthermore, we believe that data sharing works best when it leads to collaborative work. Most epidemiological studies involve complex data and valid analysis and interpretation of the data would be much more likely if those responsible for the study design and data collection are involved. In short, to enhance the quality of any further analysis, the first goal should be to involve the original investigators in the work. This is not accomplished by forcing investigators to place their data on accessible web-sites.

Thus, the prime responsibility for the ethical use of the data must surely lie with the researchers who collected it. This is particularly appropriate when the data was collected from individual study participants with prior consultation with the relevant communities. In this situation it is clearly unethical for the Principal Investigator to permit any potentially inappropriate use of data from their study. One way for ethically acceptable data sharing to occur is for the original researchers to: (i) only send data for reanalysis (or invite the other researchers to undertake a joint re-analysis) after having approved the planned work; (ii) be given the opportunity to be co-authors of any resulting publications; and (iii) if collaboration breaks down, then having the right to have a letter-to-the editor or accompanying commentary published at the same time, and in the same journal, as the reanalysis.

It is now widely recognised (e.g. in privacy legislation in many countries), that the ultimate responsibility for any particular data set (e.g. health records, cancer registrations) lies with the person(s) or agency that collected the data, and they have the responsibility to make the decisions as to who the data can or should be shared with, and for what purposes. Researchers may have an obligation to share data, under appropriate circumstances, but they also have the right and obligation to decide not to do so if the proposed uses of the data are inappropriate or unclear. Of course, in some instances researchers may inappropriately choose not to share their data, and colleagues and funding agencies may then respond appropriately, in terms of scepticism about the validity of the published work and/or lack of opportunities for further funding. So this approach is not without its difficulties. However, the alternative, i.e. compulsory data-sharing without any restriction on who can obtain a copy, has even greater difficulties, and much greater ethical and scientific concerns.

## Conclusions

In conclusion, we consider that any blanket requirement that datasets be made public after completion of a study is unethical. Obtaining prior informed consent from the participants does not solve this problem, unless the informed consent process makes it completely clear what is being proposed, in which case most study participants would not agree. Thus, we agree that "data from epidemiologic studies should be available for impartial reanalysis and reinterpretation regardless of whether the study was funded by public monies or by groups with particular interests or ideologies" [39] and that "the original authors have a responsibility to cooperate with, and facilitate, impartial and competent reanalysis and reinterpretation of their data" [39]. However, this does not remove the obligations and responsibilities that the original investigators hold for the people they invited to participate in the study.

## Abbreviations

EPA: Environmental Protection Agency; HRC: Health Research Council; MRC: Medical Research Council; NCSP: National Cervical Screening Programme; NIEHS: National Institute for Environmental Health Sciences; NIH: National Institutes of Health; OSHA: Occupational Safety and Health Administration; OECD: Organisation for Economic Co-operation and Development; STEPS: STEPwise approach to Surveillance; WHO: World Health Organisation

## Competing interests

The authors have been involved in, and been recipients of, data sharing requests.

## Authors' contributions

NP drafted the paper and AS added further material and revised the overall paper. Both authors read and approved the final manuscript.
